# Food additives and PAHO’s nutrient profile model as contributors’ elements to the identification of ultra-processed food products

**DOI:** 10.1038/s41598-023-40650-3

**Published:** 2023-08-30

**Authors:** Daniela Silva Canella, Vanessa dos Santos Pereira Montera, Natália Oliveira, Laís Amaral Mais, Giovanna Calixto Andrade, Ana Paula Bortoletto Martins

**Affiliations:** 1 Institute of Nutrition, Rio de Janeiro State University (UERJ), Rio de Janeiro, Brazil; 2 Center for Epidemiological Research in Nutrition and Health (Nupens), University of São Paulo (USP), São Paulo, Brazil; 3 Postgraduate Program in Food, Nutrition and Health, Rio de Janeiro State University (UERJ), Rio de Janeiro, Brazil; 4 Nutritional Epidemiology Observatory, Federal University of Rio de Janeiro (UFRJ), Rio de Janeiro, Brazil; 5 Brazilian Institute for Consumer Defense (IDEC), São Paulo, Brazil

**Keywords:** Diseases, Risk factors, Epidemiology

## Abstract

The NOVA classification system categorizes foods according to the extent and purpose of industrial processing. Ultra-processed food products (UPF) are frequently composed of excessive amounts of sugars, salt, oils, and fats, and cosmetic additives designed to make them palatable and/or appealing. We aimed to describe the presence of critical nutrients in excess and cosmetic additives in packaged foods and beverages and to evaluate the proportion of UPF that can be correctly identified through the presence of critical nutrients in excess or the presence of cosmetic additives in food products. A total of 9851 items available in Brazilian supermarkets containing lists of ingredients and nutrition facts panels were analyzed. Cosmetic additives and critical nutrients in excess, according to Pan American Health Organization (PAHO)’s nutrient profile model, were assessed. All food items were categorized into the four NOVA classification groups. Relative frequencies of items with at least one critical nutrient in excess and one type of cosmetic additive were estimated. For UPF, 82.1% had some cosmetic additive, and 98.8% had some cosmetic additive or a nutrient in excess. This combined criterion allowed the identification of 100.0% of sweet cookies, salted biscuits, margarine, cakes and sweet pies, chocolate, dairy beverages, and ice cream. Combining the presence of cosmetic additives and the PAHO’s nutrient profile model contributes to the identification of UPF.

## Introduction

The NOVA food classification system and the concept of ultra-processed food products (UPF) were developed in 2009^1^ and improved in 2014 with the publication of the 2nd edition of the Brazilian Dietary Guidelines^2–4^. The NOVA classification is a landmark that exposed the relevance of the extension and the purpose of industrial food processing and its relationship with diet quality and health, currently widely used in the scientific literature^5–10^ and national and international official documents^2,11–15^. The contribution of UPF to the total dietary energy is high in several countries^12^, which raises concern since the increased consumption of these products is associated with several chronic diseases, including obesity, cardiovascular diseases, diabetes, mental health, and some types of cancer^5,6,8–10^.

In this sense, the reduction of UPF consumption is essential for the improvement of population’s health. One of the key issues to achieve such reduction at a populational level is facilitating the identification of UPF by consumers, researchers, and policymakers, and for regulation purposes. In 2019, Monteiro et al*.*^3^ improved the description of elements to identify these products. Since almost all food items are processed at some level, focusing only on these criteria, such as calling these items industrialized, is not enough to distinguish them properly. UPF are formulations characterized by the utilization of ingredients for exclusively industrial use, e.g., substances that are rarely or never used in the domestic context (examples: fructose, invert sugar, interesterified and hydrogenated fats, hydrolyzed and isolated proteins), besides being composed by diverse types of food additives^3^.

Food additives have varied functions, like preservatives, antioxidants, emulsifiers, thickeners, sweeteners, and others. Some of them have the role to ensure food safety, such as preservatives^16^. However, a possible difference between UPF and other food items highlighted in the NOVA is the use of additives with cosmetic functions (henceforward called cosmetic additives), such as colors, flavors, emulsifiers, and sweeteners. These additives mask undesirable sensorial properties and give the final product special sensory characteristics, making food by-product mixtures attractive to see, taste, smell, and touch and, consequently, favoring their consumption^3,12^. The presence of cosmetic additives on foods could contribute to facilitating the identification of UPF, as this is a commonly mandatory information on the list of ingredients on food labels.

Furthermore, sugars, salt, oils, and fats, generally in combination, are frequent ingredients in the formulation of UPF^3^. Considering that, the Nutrient Profile Model (NPM) of the Pan American Health Organization (PAHO) is an efficient tool to identify processed foods and UPF that are excessive in nutrients of concern (free sugars, sodium, total fat, saturated fat, and trans fat), also called critical nutrients, or contain sweeteners^11^. The PAHO NPM is based on the World Health Organization (WHO) Population Nutrient Intake Goals to Prevent Obesity and Related Noncommunicable Diseases (NCDs)^17^ and establishes that only processed foods or UPF should be evaluated, as a position that recommends the consumption of unprocessed or minimally processed foods and freshly prepared dishes made with these foods^11^.

Contrary to what some scientific papers have been pointing out^18–20^, we hypothesize that the NOVA classification system is robust and functional, and the characteristics of foods and beverages, considering non-nutrient and nutrient profiles, such as the presence of cosmetic additives and critical nutrients in excess, can contribute to the identification of UPF and agrees with the classification of items by a trained researcher. Thus, the objective of this study was to describe the presence of critical nutrients in excess and cosmetic additives in packaged foods and beverages marketed in Brazilian supermarkets, according to the NOVA groups, and to evaluate the proportion of UPF that can be correctly identified through the presence of critical nutrients in excess or cosmetic additives in food products.

## Methods

A descriptive cross-sectional study was carried out using data from the list of ingredients and nutrition facts panel on labels of packaged foods and beverages marketed in Brazil.

The data collection took place between April and July 2017 in 10 outlets of major supermarket retail chains selling foods and beverages in two large Brazilian cities (São Paulo and Salvador). Supermarkets were chosen for the data collection since they account for the larger share of the energy acquired by Brazilian households^21^. About the cities, São Paulo is the largest city in Brazil, located in the Southeast region of the country, and Salvador is the largest city in the Northeast region. The definition of retail chains considered those with the greatest market share in Brazil. Trained researchers photographed all sides of the packaging of all the packaged foods and beverages available at the selected supermarkets^22^. Details about the sample definition and data collection are available in Duran et al*.*^23^.

Most of the unprocessed foods (such as fruits and vegetables) sold in supermarkets in Brazil are not packaged, therefore were excluded from the sample. The present study included all foods and beverages for which data from the list of ingredients and nutrition facts panel were available totaling 9,851 items.

Data from the nutrition facts panel and the list of ingredients was used to identify the items excessive in at least one of the critical nutrients (sugars, sodium, total fat, and saturated fat) or with the presence of trans fat or sweeteners, based on the thresholds of the PAHO NPM^11^ (Appendix 1), as previously described^23^. The information about total, free or added sugars was not mandatory in Brazilian food labels at the time of data collection. Among the products evaluated, only 10.8% had information on total sugars voluntarily in the nutrition facts panel. Among the products without the declaration (89.2% of the products), a method proposed by Scapin et al*.*^24^ and the Brazilian Table of Food Composition (*Tabela Brasileira de Composição de Alimentos—*TACO)^25^ was applied to estimate the content of added sugars. The recommendations from the PAHO NPM were used to estimate the content of free sugars^11^. Additionally, information from the list of ingredients was used to identify the presence of sweeteners and trans fat-source ingredients (such as 'hydrogenated vegetable fat', 'partially hydrogenated fat', 'hydrogenated vegetable oil', and 'hydrogenated')^26^. The PAHO NPM was only applied to processed foods or UPF.

Food additives described in the list of ingredients on food labels were first identified as defined by the National Health Surveillance Agency (*Agência Nacional de Vigilância Sanitária—*Anvisa), which sets out the types of additives allowed by food category, and grouped according to their function such as preservatives, antioxidants, emulsifiers, thickeners, and sweeteners, among others^16^. Then, the additives were categorized as cosmetic or not cosmetic, considering the proposition of the NOVA classification^3^. Cosmetic additives include flavors, flavor enhancers, colors, emulsifiers, emulsifying salts, sweeteners, thickeners, anti-foaming, bulking, carbonating, foaming, gelling, and glazing agents^3^. Some food additives may have more than one function. In this case, it was considered the function most commonly described in other foods of the same category and which were authorized by Anvisa for the category of foods in question. The process was conducted by a trained researcher (VSPM), as described in Montera et al*.*^27^. As sweeteners are included as a critical nutrient in the PAHO NPM and are also a cosmetic additive, in our study we considered their presence only when we applied the PAHO NPM, in order to not overestimate its frequencies.

All food items were independently classified into the groups and subgroups of the NOVA by a trained researcher (GCA), with experience in studies related to the classification, into unprocessed or minimally processed foods; processed culinary ingredients; processed foods; and UPF^3^. Definitions of the NOVA food groups are available in Appendix 2.The relative frequencies (%) of processed foods and UPF and their subgroups with critical nutrients in excess based on the PAHO NPM were estimated. The proportion of items, considering the four groups of the NOVA, with the presence of at least one type of cosmetic additive and of only flavors or colors, which were the most used additives^27^, were also estimated. Additionally, the proportion of processed foods and UPF with the presence of at least one cosmetic additive or excess of one or more critical nutrients was assessed.

Finally, the agreement between the classification of UPF made by a trained researcher and the combination of the presence of cosmetic additives or the excess of critical nutrients was assessed using the kappa agreement coefficient and the percentage agreement. The kappa coefficient was interpreted using Landis & Koch (1977)^28^: below 0.00—poor; 0.00–0.20—slight; 0.21–0.40—fair; 0.41–0.60—moderate; 0.61–0.80—substantial; and 0.81–1.00—almost perfect.

## Results

Among the 9,851 items assessed, 71.9% were classified, by a trained researcher, as UPF, 13.7% as processed foods, 12.0% as unprocessed or minimally processed foods, and 2.4% as processed culinary ingredients. Of the total, 96.1% of the processed foods or UPF had some critical nutrient in excess, 60.2% of the products had at least one cosmetic additive and 52.1% had flavors or colors (data not shown in table).

Regarding critical nutrients, 97.1% of the UPF and 90.9% of the processed foods had one or more nutrients of concern in excess. Considering the presence of cosmetic additives in foods according to the NOVA groups, 82.1% of the UPF had at least one cosmetic additive, while among processed foods, processed culinary ingredients, and unprocessed or minimally processed foods the percentual was 3.7%, 0.4%, and 5.1%, respectively. Taking into account only the presence of flavors or colors, those were presented in 71.4% of the UPF. Among the UPF, 98.8% had one or more cosmetic additives or presented some critical nutrient in excess (Table 1).

**Table 1:** Proportion (%) of food items available in Brazilian supermarkets with cosmetic additives and/or critical nutrients in excess based on the PAHO NPM, according to the NOVA classification system carried out by a trained researcher.

NOVA food groups and subgroups	Total sample		Presence of (%)			
	n	(%)	At least one critical nutrient in excess*	At least one cosmetic additive	Flavors or colors	At least one critical nutrient in excess or cosmetic additive*
**Unprocessed or minimally processed foods**	1,178	12.0	–	5.1	4.9	–
Milk	154	1.6	–	0.6	–	–
Pasta	144	1.5	–	38.2	38.2	–
Others^1^	213	2.2	–	1.9	1.4	–
**Processed culinary ingredients**	237	2.4	–	0.4	0.4	–
Animal fats	38	0.4	–	2.6	2.6	–
**Processed foods**	1,352	13.7	90.9	3.7	1.4	89.1
Bread	60	0.6	83.3	1.7	1.7	83.3
Cheese	226	2.3	99.1	0.4	–	98.7
Salted/dried/smoked meat	88	0.9	98.9	1.1	–	98.9
Others^2^	978	9.9	88.7	4.8	1.8	86.4
**Ultra-processed food products**	7,084	71.9	97.1	82.1	71.4	98.8
Cold cuts and sausages	611	6.2	99.5	88.7	76.3	99.7
Sweet cookies	602	6.1	99.7	93.4	89.5	100.0
Salted biscuits	459	4.7	99.1	80.6	66.2	100.0
Margarine	53	0.5	100.0	94.3	92.5	100.0
Cakes and sweet pies	247	2.5	100.0	95.5	91.5	100.0
Bread	283	2.9	96.8	71.4	22.6	98.2
Sweets in general	1,232	12.5	97.6	75.8	68.4	98.6
Carbonated beverages	105	1.1	95.1	95.2	95.2	99.0
Chocolate	233	2.4	100.0	98.3	91.0	100.0
Pizza, lasagna or pastry	356	3.6	94.9	69.9	56.7	97.2
Ready meals	387	3.9	95.8	69.5	59.7	97.7
Other sugary drinks	767	7.8	87.4	90.4	86.2	96.3
Dairy beverages	451	4.6	99.8	96.0	83.6	100.0
Ice cream	240	2.4	100.0	93.8	84.6	100.0
Sauces and spreads	632	6.4	97.3	81.5	64.6	98.9
Others^3^	381	3.9	99.5	53.5	45.4	99.2

^1^Coffee, tea, seafood, meat from other animals, nuts and seeds, dried fruits and vegetables, Brazilian typical meals, and other ready meals.

^2^Salted/dried fish, dried seafood, canned fish, eggs, vegetables, pulses or cereals, olives, salted peanuts, fruit-based sweets, and seasonings.

^3^Tablets (condiments) and ready-made seasonings, ultra-processed cheeses, and breakfast cereals.

*Only applied to processed foods and ultra-processed food products.

It was verified substantial agreement between the classification of UPF made by the trained researcher and the presence of some cosmetic additive (k = 0.6933; 86.0%, data not shown) and with the combination of cosmetic additives or some critical nutrient in excess (k = 0.6015; 86.0%, data not shown in table).

The identification of some critical nutrient in excess among UPF subgroups was more frequently in margarine, cakes and sweet pies, chocolate, and ice cream (100.0%), sweet cookies (99.7%), and cold cuts and sausages (99.5%). On the other hand, only 87.4% of other sugary drinks had some critical nutrient in excess. Regarding the UPF subgroups, those that more frequently presented at least one cosmetic additive were: chocolates (98.3%), dairy beverages (96.0%), cakes and sweet pies (95.5%), carbonated beverages (95.2%), and margarine (94.3%), while bread, pizza, lasagna or pastry, and ready meals were those that less frequently had cosmetic additives (71.4%, 69.9% and 69.5%, respectively). Considering the criterion combination (presence of cosmetic additive or critical nutrient in excess), it was possible to identify 100.0% of the sweet cookies, salted biscuits, margarine, cakes and sweet pies, chocolate, dairy beverages, and ice cream, 99.7% of cold cuts and sausages, and 99.0% of carbonated beverages previously classified as UPF. It also identified 97.7%, 97.2%, and 96.3% of ready meals, pizza, lasagna or pastry, and other sugary drinks, respectively (Table 1).

## Discussion

In the present study, using data from almost 10,000 packaged foods and beverages marketed in Brazilian supermarkets, we verified a high frequency of cosmetic additives in UPF, compared to other NOVA groups. The information from the list of ingredients and the nutrition facts panel of foods and beverages analyzed in this study, such as the characteristics related to non-nutrient and nutrient profiles, following a rigorous model as PAHO’s, was useful, presented a substantial agreement to discriminate NOVA groups, and allowed the proper identification of most UPF.

The option to focus on cosmetic additives and critical nutrients in excess in foods and beverages in our analyses has three main reasons: 1) these characteristics, in some way, are currently mandatorily available on food labels, in Brazil and other countries; 2) their presence indicates an unfavorable non-nutrient and nutrient composition, which contributes to the hyper-palatability, favors the overconsumption of UPF and is associated with negative health outcomes; 3) the combined identification of food additives and critical nutrients in excess is convenient for the recognition of UPF since some items, such as diet beverages, can escape from regulatory measures only related with nutrients, such as the front-of-package nutrition labeling (FoPNL). The proper and clear presentation of these elements in labels can be a regulatory strategy to better inform the population about UPF and contribute to the prevention of NCDs.

An increasing trend in the use of cosmetic additives has been observed worldwide. Considering the period from 2006 to 2019, the annual increase rate in the use of these industrial ingredients among low-, middle-, and high-income countries was estimated at 7.0%, 2.9%, and 4.8%, respectively^29^. Additionally, studies have shown a considerable frequency of cosmetic additives in foods and beverages in different countries^27,30–32^. In France, a study assessed the distribution of food additives in 106,000 items and identified that, among the three most frequent additives, emulsifiers and starch modifiers are considered cosmetic additives and only citric acid is a preservative^30^. In Brazil, one research using the same dataset as this study evaluated the distribution of food additives in 9,856 items and found that among the five most prevalent additives, only preservatives were not cosmetic additives, while flavors, colors, stabilizers, and emulsifiers were present in 47.1%, 27.8%, 27.6%, and 19.4% of the sample, respectively^27^.

In our study, the evaluation of nutrients of concern was based on the PAHO NPM, which aims to define and identify processed foods and UPF with critical nutrients in excess associated with NCDs, according to the goals established by WHO. The model was developed to be applied in policies for the reduction in the supply and demand of these unhealthy products in food environments^33^. Studies conducted in Brazil and Australia have shown that this nutrient profile model is able to better identify UPF as eligible to receive a warning as a FoPNL than other models in the classification of food items with excessive amounts of critical nutrients^23^ or as healthy/unhealthy^34^. Indeed, the PAHO NPM has been adopted as a reference for the implementation of FoPNL in the format of warnings in Latin America, including Mexico, Argentina, and Colombia^35–37^. A study conducted in Australia analyzing the dietary intakes of Australians aged 2 + years showed that the majority of the participants consumed daily at least three processed foods and UPF with excessive amounts of critical nutrients, according to the PAHO NPM. These same participants had higher intakes of free sugars, total fats, saturated fats, trans fats and sodium than those not consuming these products^38^. In this way, two Uruguayan studies with children investigated the consumption of foods and beverages according to the PAHO NPM. The one with school-aged children of the School Feeding Program showed that 25% the children consumed four or more products identified as excessive in free sugars, total fats or saturated fats by PAHO NPM; in the case of excessive sodium it was 40%^39^. And the other with children from two to four years old showed that 50% of them consumed three or more products excessive in some critical nutrient (free sugars, total fats, saturated fats and sodium), and 90% of the participants consumed products consumed in at least one of the critical nutrients^40^. A study conducted with representative samples of nine countries in the Americas estimated the effects of the consumption of foods above the PAHO NPM thresholds on diet quality. The conclusion was that dietary patterns with one or more UPF and processed foods with an excess of critical nutrients according to PAHO NPM were directly associated with the excessive consumption of nutrients that constitute a risk for NCDs and, therefore, are associated with an unhealthy diet in the region^33^.

According to our results, the strategy of identifying items with cosmetic additives combined with excessive amounts of critical nutrients seemed to be useful in the correct classification of UPF. If the Brazilian FoPNL used an adequate nutrient profile model and included information on cosmetic additives, it could support individuals making healthier food choices by identifying almost all UPF.

The difference between pizza, lasagna and pastry, ready meals, and other UPF subgroups, which were the items with a lower presence of cosmetic additives, is remarkable. Those subgroups, which represent about 1.5% of total energy consumed in Brazil^41^, are complex to classify in dietary surveys, since they could be a culinary preparation based on unprocessed or minimally processed foods or a UPF, being additional information (ex: label, brand, who prepared) important to its proper classification. Furthermore, those are examples of UPF that could have hidden additives, as highlighted in qualitative analysis^42^. This is because the list of ingredients of these items often includes other processed and UPF, called “compound ingredients”, which is allowed by the Brazilian regulation, but may contain food additives in their composition. Therefore, there are food additives in the final product to which consumers are exposed, but these are not described in the list of ingredients, as they were not declared directly on food labels, giving the impression that the final product does not contain or contains little food additives. These aspects point out a need to improve Brazilian labels, especially the list of ingredients and sugars information, in order to facilitate the identification of UPF through the label and estimulate better food choices by the consumers.

Another notable result is that more than 1/3 of minimally processed pasta had cosmetic additives, which is a result of the presence of colorings in these items. However, this inclusion does not have the intention to make the final product palatable or more appealing, so it should not transform them into UPF. To exemplify this situation, we present a list of ingredients of one pasta available in our dataset: wheat flour enriched with iron and folic acid, egg white, eggs, and natural colorings urucum and turmeric.

Braesco et al*.*^19^ assessed the consistency in the food assignments in the NOVA groups by specialists using two lists: one with 120 marketed food products with ingredients information and another with 111 generic food items from dietary surveys without ingredients information. In the marketed foods list, more similar to our dataset, the study focused on three categories of foods: fresh dairy products, bread products, and mixed dishes. According to the authors, the evaluators were specialists (researchers with expertise in food technology and human nutrition, health professionals, and food industry professionals) and received the same information about the NOVA classification system. A fair agreement (mean values of Fleiss’ κ = 0.32) was found between the evaluators and the professional background did not affect consistency among evaluators. On the other hand, in our study, for UPF we found a substantial agreement (k = 0.6015) between a trained evaluator’s classification and the combined criterion of cosmetic additives or critical nutrients in excess. It is worth mentioning that 71.4% of bread previously classified as UPF presented cosmetic additives and if we also considered the critical nutrients, 98.2% of them would be identified, while ready meals classified as UPF had cosmetic additives in 69.5% of the cases and also critical nutrients in 97.7%.

Despite being specialists in human nutrition and/or in food technology and having the information about the NOVA classification, the selection of the professionals in the study of Braesco et al*.*^19^ is poorly described, and their training and experience concerning the application of the NOVA classification were not mentioned, which can contribute to the misclassification observed. One example that can corroborate this was observed in the generic foods list: commercial orange juice was classified by specialists as unprocessed or minimally processed food in 16.0% of the assignments, as a processed culinary ingredient in 11%, as processed food in 34.5%, and as UPF in 38.5% of the assignments^19^. Considering the NOVA classification system^3^, it is impossible to classify this item as a processed culinary ingredient, which can reveal the low familiarity of specialists with the NOVA classification.

In dietary surveys, food frequency questionnaires (FFQ) cannot consider the NOVA in their development and 24-h diet recalls or diet records cannot collect detailed information about food items. Seeking the improvement in the reproducibility of data analyses, Khandpur et al*.*^43^ applied a four-stage classification approach to three different surveys conducted in the United States (US), using FFQ. The approach included: (i) creating a complete list of food items from the FFQ; (ii) assigning food items to a NOVA group by three researchers; (iii) checking for consensus in categorization and identifying discordant items; and (iv) discussing with experts and using of additional resources to guide the final categorization of the discordant items. Other studies have documented approaches used in the classification of food items using the NOVA^44,45^.

Another important aspect is the comprehension of food groups by the population, since the NOVA has been used in the recommendations of different dietary guidelines, for example. A qualitative study conducted with Brazilian adults found that participants seemed to understand the NOVA classification regarding food processing and additives used, and that they easily identified its extreme groups: unprocessed or minimally processed foods and UPF^46^.

The NOVA classification considers the extension and the purpose of industrial food processing and that the UPF are industrial formulations characterized (also, but not only) by the utilization of some specific cosmetic food additives, and that they are typically high energy-dense food products, high in sugars, unhealthy fats, and sodium. According to Monteiro et al*.*^3^, a practical way to identify an UPF is to check if its list of ingredients contains at least one item characteristic of the NOVA UPF group (markers of UPF). For the population, but also for dietary surveys, this practical strategy is useful, as shown in our study, due to the availability of information from lists of ingredients and critical nutrients (or the possibility to include them in labels), different from the information of industrial processes and their purpose, which food manufacturing industry is not obliged to present. Considering these elements, despite the debate in the literature, we believe that the NOVA classification system and the concept of UPF are valid to be used in scientific research and public policies, such as dietary guidelines^14^.

This study has some limitations. It did not cover all packaged foods and beverages available in the Brazilian retail market, including specific regional items not widely available in the country. However, supermarkets, our setting, are responsible for more than half of total food energy and UPF energy purchased in Brazil^21^. Additionally, we did not consider other markers of UPF, such as substances that are rarely or never used in domestic kitchens, but we focus on those with more evidence related to health outcomes^11,47^.

Despite these limitations, the present study involved a comprehensive assessment of the occurrence of cosmetic additives or excessive amounts of critical nutrients in almost 10,000 packaged foods and beverages available in a middle-income country's retail market. To our best knowledge, no study without conflicts of interest tested if cosmetic additives or critical nutrients in excess are capable of adequately classifying UPF. As the NOVA classification has been used as a conceptual reference for public policies in different countries, we believe that this study could highlight the strength and potential of the NOVA classification and be an important contribution to food policies and nutrition research.

In conclusion, the presence of cosmetic additives combined with the application of the PAHO’s nutrient profile model to identify critical nutrients in excess is consistent in the identification of UPF. The adoption of these two characteristics, related to non-nutrient and nutrient profiles, or its improvement in labels, like in the FoPNL, could contribute to the recognition of UPF by the population.

### Supplementary Information

Supplementary Information.

## Data Availability

The data used in this article are available upon reasonable request directed to LAM (lais.amaral@idec.org.br).
