# Supplementary material for: Food additives and PAHO’s nutrient profile model as contributors’ elements to the identification of ultra-processed food products
Source: Sci Rep. 2023 Aug 30;13:13698. doi: 10.1038/s41598-023-40650-3 (PMC10468485; doi:10.1038/s41598-023-40650-3)
Supplement: Supplementary file 1 — Supplementary Information. [file 41598_2023_40650_MOESM1_ESM.doc]

**SUPPLEMENTARY INFORMATION**

**Appendix 1**

PAHO Nutrient Profile Model criteria for identifying processed and ultra-processed food products excessive in sodium, free sugars, other sweeteners, saturated fat, total fat, and trans fat.

| **Sodium** | **Free sugars** | **Other sweeteners** | **Total fat** | **Saturated fat** | **Trans fat** |
| --- | --- | --- | --- | --- | --- |
| > 1 mg of sodium per 1 kcal | > 10% of total energy from free sugars | Any amount of other sweeteners | > 30% of total energy from total fat | > 10% of total energy from saturated fat | > 1% of total energy from trans fat |

Source: PAHO (2016).

**Appendix 2**

Definition of the NOVA food groups, according to the extent and purpose of food processing.

| **NOVA groups** | **Definition** |
| --- | --- |
| **Group 1 -Unprocessed or minimally processed foods** | Unprocessed: edible parts of plants (fruits, seeds, leaves, stems, roots, tubers) or of animals (muscle, offals, eggs, milk), and also fungi, algae and water, after separation from nature.  Minimally processed: unprocessed foods altered by industrial processes such as removal of inedible or unwanted parts, drying, crushing, grinding, fractioning, roasting, boiling, pasteurisation, refrigeration, freezing, placing in containers, vacuum packaging, non-alcoholic fermentation, and other methods that do not add salt, sugar, oils or fats or other food substances to the original food. The main aim of these processes is to extend the life of unprocessed foods, enabling their storage for longer use, and, often, to make their preparation easier or more diverse. Infrequently, minimally processed foods contain additives that prolong product duration, protect original properties or prevent proliferation of microorganisms. |
| **Group 2 -Processed culinary ingredients** | Substances obtained directly from group 1 foods or from nature by industrial processes such as pressing, centrifuging, refining, extracting or mining. Their use is in the preparation, seasoning and cooking of group 1 foods. These products may contain additives that prolong product duration, protect original properties or prevent proliferation of microorganisms. |
| **Group 3 -Processed foods** | Products made by adding salt, oil, sugar or other group 2 ingredients to group 1 foods, using preservation methods such as canning and bottling, and, in the case of breads and cheeses, using non-alcoholic fermentation. Processes and ingredients here aim to increase the durability of group 1 foods and make them more enjoyable by modifying or enhancing their sensory qualities. These products may contain additives that prolong product duration, protect original properties or prevent proliferation of microorganisms. |
| **Group 4 -Ultra-processed food products** | Formulations of ingredients, mostly of exclusive industrial use, that result from a series of industrial processes (hence ‘ultra-processed’), many requiring sophisticated equipment and technology. Processes enabling the manufacture of ultra-processed foods include the fractioning of whole foods into substances, chemical modifications of these substances, assembly of unmodified and modified food substances using industrial techniques such as extrusion, moulding and pre-frying, frequent application of additives whose function is to make the final product palatable or hyper-palatable (‘cosmetic additives’), and sophisticated packaging, usually with synthetic materials. Ingredients often include sugar, oils and fats, and salt, generally in combination; substances that are sources of energy and nutrients but of no or rare culinary use such as high fructose corn syrup, hydrogenated or interesterified oils, and protein isolates; cosmetic additives such as flavours, flavour enhancers, colours, emulsifiers, sweeteners, thickeners, and anti-foaming, bulking, carbonating, foaming, gelling, and glazing agents; and additives that prolong product duration, protect original properties or prevent proliferation of microorganisms. Processes and ingredients used to manufacture ultra-processed foods are designed to create highly profitable products (low cost ingredients, long shelf-life, emphatic branding), convenient (ready-to-consume) hyper-palatable snacked products liable to displace all other NOVA food groups, notably group 1 foods. |

Source: Monteiro et al (2019).
